# Electroformation of Giant Vesicles on Indium Tin Oxide (ITO)-Coated Poly(ethylene terephthalate) (PET) Electrodes

**DOI:** 10.3390/membranes1020109

**Published:** 2011-05-26

**Authors:** Yukihisa Okumura, Yuuichi Iwata

**Affiliations:** Department of Chemistry and Material Engineering, Faculty of Engineering, Shinshu University, 4-17-1 Wakasato, Nagano 380-8553, Japan; E-Mail: yiwata@mc84.shinshu-u.ac.jp

**Keywords:** electroformation, electroswelling, giant vesicles, giant liposomes, lipid membrane

## Abstract

Electroformation of cell-sized lipid membrane vesicles (giant vesicles, GVs) from egg yolk phosphatidylcholine was examined using a poly(ethylene terephthalate) sheet coated with indium tin oxide (ITO-PET) as the electrode material. With sinusoidal ac voltage, GV formation occurred in a similar manner to that on an ITO-glass electrode widely used in electroformation. Difference in the specific electrical resistance of ITO-PET did not significantly affect electroformation. The present results indicate that ITO-PET may be used as more flexible and less expensive electrode material in electroformation. In order to obtain insights into electroformation, other electric voltage forms, static dc and dc pulses, were also tested in place of commonly used sinusoidal ac. Under the present conditions, the best GV formation was observed with dc pulses of negative polarity. The result with static dc demonstrated that the mechanical vibration of swelling lipid seen with sinusoidal ac voltage was not essential for GV formation. On the positive electrode, the electroswelling of lipid mainly yielded non-spherical membranous objects. Pre-application of positive dc voltage on lipid hindered GV formation in electroswelling of the lipid with ac.

## 1. Introduction

Large lipid membrane vesicles of the size comparable with biological cells (giant vesicles, GVs) have been used in various studies as model membranes [1,2]. Among the known preparation methods of GVs [2], swelling of lipid on an electrode upon application of electric voltage, commonly known as electroformation or electroswelling, has been widely used [2,3]. The method is relatively simple and may yield many large GVs (typically 10–100 μm in diameter) with reproducible results under various conditions including physiologically relevant ones [4,5].

A pair of thin wire electrodes made of platinum or a set of two planar electrodes have been the two different electrode setups of electroformation commonly used [6,7,8,9]. The latter setup can produce more vesicles at one time because of the larger surface area. It is also possible to modify the structure of the setup, for example, to a flow chamber or microfluidic channels [10,11].

For the material of the planar electrodes, almost all the previous studies used electroconductive glass coated with indium tin oxide (ITO-glass) because its transparency to light allows observation with an optical microscope [9,10,11,12,13,14]. ITO-coated poly(ethylene terephthalate) (ITO-PET) sheets are also commercially available and used as electrode materials in organic electroluminescence. ITO-PET has a few advantages over ITO-glass. It is more flexible and easily cut to arbitrary shapes. This allows more variation in the design of electroformation chamber. ITO-PET is soft and can tolerate a stronger physical shock than ITO-glass of the same thickness. It is also less expensive. ITO-PET might be suitable for the mass production of small disposable electroformation chambers. With electroformed GVs on electrode, such a chamber could have an application, for example, in a sensor device for clinical use. However, to the best of our knowledge, electroformation on this material has not yet been studied.

In the present study, electroformation on ITO-PET sheets was examined. In addition to commonly used sinusoidal ac, static dc and dc pulses were tested as applied voltage, to obtain some valuable insights into electroformation.

## 2. Results and Discussion

### 2.1. Electroformation Chamber with ITO-PET Sheet Electrode

**Figure 1:**
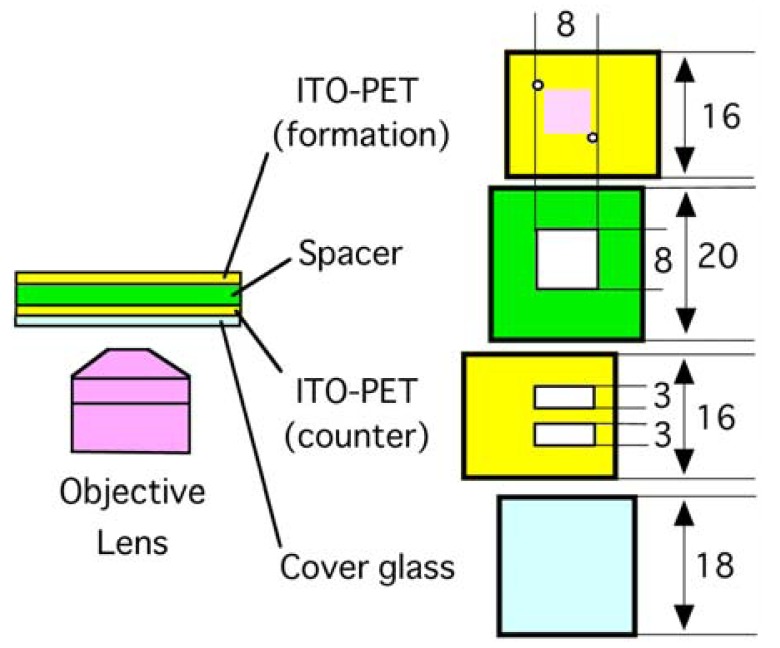
An electroformation chamber with two ITO-PET electrodes. Lipid (shown as a pink square) was deposited on the formation electrode. To make observation with an inverted optical microscope possible, two slits were made in the counter electrode. The unit of length is millimeter.

A typical electroformation chamber with ITO-glass electrodes has two pieces of ITO-glass plates facing each other in parallel and separated by a spacer [9]. In this study, a similar chamber was constructed using ITO-PET sheets as the electrode material as schematically illustrated in Figure 1. However, simple use of intact ITO-PET sheets was not suitable for microscopic observation. Looking through the ITO-PET sheet, only a blurred image of the interior of the formation chamber could be seen. This is probably because the anisotropic nature of the used PET material could interfere with the optical system in the present study. To make observation with the inverted optical microscope possible, a part of the bottom counter electrode was removed to make windows. Over the windows, a piece of thin cover glass was attached. Also, two small holes were made in the formation electrode to facilitate the introduction of ultrapure water into the chamber. These modifications of ITO-PET sheets were made without difficulty by using a small knife and a drill. The softness and flexibility of ITO-PET allows such easy processing.

### 2.2. Electroformation on ITO-PET with Sinusoidal ac Voltage

**Figure 2:**
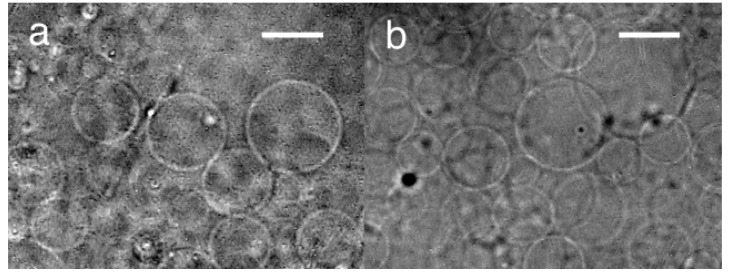
**(a)** Electroformation of GVs on an ITO-PET electrode with sinusoidal ac voltage. The image was taken at 160 min after the application of the ac (3.0 Vpp, 2 Hz); **(b)** Electroformation on ITO-glass under the comparable conditions. Bar = 20 μm.

Upon application of sinusoidal ac voltage (3.0 Vpp (peak-to-peak voltage of 3.0 V), 2 Hz), the deposit of egg phosphatidylcholine (eggPC) on the formation electrode (specific electrical resistances, 0.4 Ωm) started to swell and vibrated in synchronization with the applied voltage. The completion of the formation took 90–160 min. Many spherical GVs of approximately 20 μm in diameter were seen at the top of the swelled lipid layer, which was located at approximately 20–40 μm from the surface of the ITO-PET electrode (Figure 2(a)). Some GVs were as large as 40 μm.

In the present study, GV formation was evaluated by determining the percentage of the area on the lipid deposit covered with GVs (GV formation index). Also, the approximate thickness of the swelled lipid layer (the distance between the center of GVs sitting on the top of the swelled film of lipid and the electrode surface at the end of electroformation) was also estimated based on the focal distance [9].

Three ITO-PET sheets of different specific electrical resistances were tested with various voltages, and the results were shown in Table 1. The inferior GV formation was seen on the ITO-PET of the high specific electrical resistance at the low voltage. Other than that, similar electroformation was observed for all the cases.

**Table 1:** GV formation on ITO-PET electrodes with sinusoidal ac voltage (2 Hz).

**Specific electrical resistance [Ωm]**	**Applied voltage [Vpp]**	**GV formation index [%]**	**Typical diameter of GVs formed at the top of the lipid layer [μm]**	**Thickness of the swelled lipid layer[μm]**
0.4	3.0	20	20	20–40
	5.0	30	20	30–40
	7.0	20	20–40	30–60

0.6	3.0	10	20–30	20–50
	5.0	20	10–30	10–40
	7.0	20	20	20–40

1.0	3.0	0.5	30	30
	5.0	10	10–20	10–40
	7.0	20	20–40	20–50

For comparison, electroformation on an ITO-glass electrode was also tested. The GV formation was similar to that with ITO-PET under comparable conditions (Figure 2(b)) and occurred as previously reported by others [9]. The GVs (20–40 μm) were found at the top of the lipid layer (thickness 10–40 μm) with a GV formation index of 30%.

### 2.3. Electroformation with Static dc Voltage

**Figure 3:**
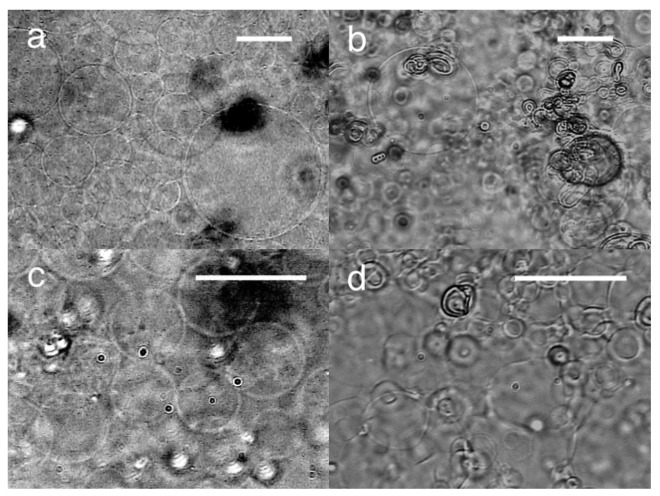
**(a)** Electroformation of GVs on the ITO-PET electrode of negative polarity. The image was taken at 120 min after the application of static dc voltage (3.0 V); **(b)** Swelled lipid on the positive electrode (3.0 V) after 150 min; **(c)** GVs formed by electroswelling with ac (5.0 V, 2 Hz) for 90 min after the pre-application of negative static dc voltage (1.5 V) for 120 min; **(d)** Distorted spherical objects formed by the pre-application of positive static dc voltage (3.0 V) for 1 min before electroswelling with ac (5.0 V, 2 Hz) for 90 min. Bar = 40 μm.

Electroformation with static dc voltage was also tested. At 3.0 V, GV formation from the lipid deposit was observed on the negative (ground) electrode. The formation was similar to that with ac voltage. The typical diameter of the GVs was 40–60 μm (Figure 3(a)). Some GVs were larger than 100 μm. The GV formation index was 50%, which was better than ac (3.0 Vpp, 2 Hz). Although it has been suggested that the mechanical vibration of swelling lipid under ac voltage could assist GV formation [3], the present results indicate that the effect should be marginal, if any.

In the first 5 min after the application of the static dc, the swelling lipid on the negative electrode showed considerable fluctuation. The fluctuating membranous structure was unstable, and frequent morphological transformation of those structures was seen. Interruption of the electric voltage at this point gradually moved the swelled lipid toward the electrode surface, indicating that the lipid had been forced to be away from the electrode under the applied voltage. Leaving the swelled lipid without any voltage for 90 min produced irregular membranous objects without GV formation. On the other hand, the following application of sinusoidal ac voltage (5.0 Vpp, 2 Hz) immediately after the interruption of the initial static dc yielded GVs as usual.

When the polarity of the formation electrode was set positive, irregular membranous structure was mostly seen. GV formation was observed but sporadic (Figure 3(b)). Angelova and Dimitrov studied electroformation with static dc voltage on a thin platinum wire electrode in their pioneering works of electroformation [7,15]. Their results, GV formation similar to ac on negative electrode and poor electroswelling on positive one, are consistent with our present observation with planar ITO-PET electrodes.

At 1.5 V, the lipid swelling on the negative electrode was much less extensive than at 3.0 V, and no GV formation occurred. The application of ac voltage (5.0 Vpp, 2 Hz) on the lipid at this point yielded GVs on the previously negative electrode (Figure 3(c)), indicating that the lipid layer was still capable of GV formation. Apparently, the static dc voltage of 1.5 V was too low to drive electroformation. Together with the experiment in which the applied dc static voltage was interrupted after 5 min, the result indicates that the pre-application of negative voltage has little effect on the lipid and the following electroformation of GVs. In contrast, when positive static voltage (3.0 V) was applied for 1 min before electroswelling with ac (5.0 Vpp, 2 Hz) for 120 min, the formation of distorted spherical objects was observed (Figure 3(d)). The effect of the pre-application of static dc voltage on lipid was different depending on the polarity. The positive voltage seemed to have a strong effect on the nature of the lipid layer. This suggests the presence of a certain internal lipid structure that should be necessary for GV formation but was destroyed upon the application of positive voltage.

The application of static dc voltage higher than 5 V sometimes turned a part of the ITO electrode surface brown. At the higher voltage, the discoloration was more often and extensive. Although this did not seem to affect electroformation significantly, the transparency of the electrode to visible light decreased. The change was not observed with sinusoidal ac below 10 V. Similar discoloration was also observed with ITO-glass, and this is probably due to an electrochemical reaction involving ITO. Since sufficient GV formation can occur with the voltage around 3 V, this does not cause a problem in using ITO-PET for usual electroformation.

### 2.4. Electroformation with dc Pulses

**Figure 4:**
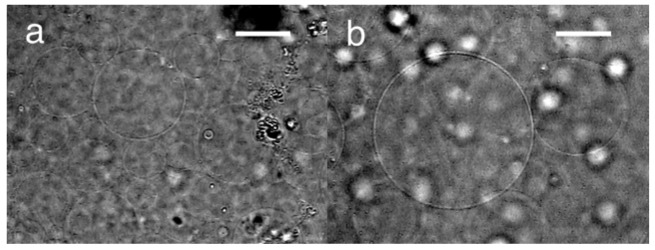
**(a)** Electroformation of GVs on the electrode of negative polarity with dc pulses; **(b)** A GV larger than 100 μm. The images were taken at 150 min after the application of dc pluses (3.0 V, 2 pulses/s, 50% duty). Bar = 40 μm.

Upon application of dc pulses (3.0 V, 2 pulses/s, 50% duty), the deposited lipid layer on the negative electrode started to swell, vibrating in synchronization with the switching of the voltage. The lipid layer was tensed when the voltage was on and relaxed during the off period. Electroformation occurred in a similar manner to that with ac. After 100 min, spherical GVs were observed on the negative electrode (Figure 4). The typical diameter of the GVs that were formed at the top of the swelled lipid layer was 50–60 μm, which was larger than ac (20–40 μm). The GV formation index was 80%, and this is also higher compared with ac (20%). The characteristics of the typical GV formation with the three forms of electric voltage, ac, static dc and dc pulses, under the comparable conditions were summarized in Table 2.

**Table 2:** GV formation on ITO-PET electrodes (0.4 Ωm) with different forms of electric voltage.

**Applied Voltage**	**GV formation index [%]**	**Typical diameter of GVs formed at the top [μm]**	**Thickness of swelled lipid layer [μm]**
Sinusoidal ac 3.0 Vpp, 2 Hz	20	20–40	20–70
Static dc 3.0 V, negative	50	40–60	90–130
Dc pulses 3.0 V, 2 pulses/s, negative	80	50–60	60–70

Under the conditions of the present study, the best electroformation of GVs was seen with the dc pulses. Compared with sinusoidal ac, the generation of dc pulses requires a less complicated electrical circuit, and this may be an advantage in application. Also, peroxidation of lipid during electroformation was previously reported [16] although no oxidized lipid was detected by thin layer chromatography after electroformation with ac voltage in the present study. The electrochemical oxidation should occur on a positive electrode [16]. Electroformation on a negative electrode with dc voltages could prevent possible oxidation. This may be another advantage of using dc.

With the static dc, the swelling lipid was continuously pulled toward the opposite electrode without the relaxation. This could result in the thicker layer.

To optimize the GV formation, the electroformation with dc pulses on the negative electrode was further examined with various pulse rates, and the results were summarized in Table 3. The optimal rate of the pulses under the conditions described was found to be 2 pulses/s. At 10 pulses/s, the GV formation index became lower, and the diameter of the GVs was also smaller. At this pulse rate, the vibration of the lipid layer was small and barely visible. Although GV formation does not necessarily require mechanical vibration as demonstrated by the present study, the small vibration suggests that the electrokinetic phenomenon underlying electroformation could not immediately respond to the rapid switching of the applied electric voltage. This could cause the attenuation of the electrokinetic driving and the deterioration of GV formation.

**Table 3:** GV formation on ITO-PET (0.4 Ωm) with negative dc pulses (3.0 V, 50% duty).

**Pulse rate [s^−1^]**	**GV formation index [% ]**	**Typical diameter of GVs formed at the top [μm]**	**Thickness of swelled lipid layer [μm]**
1	60	30–50	40–70
2	80	50–60	60–70
4	70	30–50	50–80
10	30	20–40	50–70

With dc pulses of higher voltage (5.0 V), the stronger vibration and significantly larger swelling of lipid were observed. However, this did not necessarily result in efficient GV formation. At 10 min, the top part of the swelled lipid layer reached approximately 600 μm apart from the electrode surface and evidently was detached from the rest of the swelled lipid remaining on the electrode surface. This part of the lipid turned into a mass of irregular membranous structure floating far from the electrode. Our previous study showed that GVs could be formed from a lipid deposit on a substrate that was placed between two parallel electrodes [17]. However, even though the lipid mass was present between the electrodes, no GV formation occurred. This suggests that attachment of lipid onto an immobile object, either an electrode or a substrate, is essential for GV formation.

Among the remainder of the swelled lipids near the electrode surface, the lipids located 80 μm from the electrode surface also became irregular membranous objects, and most of GVs formed at 50 μm. By the end of the electroformation, the GVs near the electrode were covered and contaminated by the irregular membranous objects.

## 3. Experimental Section

### 3.1. Materials

Phosphatidylcholine extracted and purified from egg yolk was obtained from Avanti Polar Lipids (Alabaster, AL, USA). The purity of the phospholipids was checked with thin layer chromatography on a silica gel plate (Silicagel 70 Plate-Wako from Wako Pure Chemicals (Osaka, Japan)) developed by a mixture of chloroform, methanol and water (65:25:4 v/v/v) as the solvent, and only a single spot was detected. ITO-PET sheets (nominal surface resistances of 40–45 Ω (cat# 63931-1), 50–70 Ω (cat# 63930-3) and 90–110 Ω (cat# 63928-1)) and ITO-glass were purchased from Sigma-Aldrich (St. Louis, MI, USA) and Yamakyu Special Glass (Tachikawa, Tokyo, Japan), respectively. Methanol was of the analytical grade and a product of Wako Pure Chemicals.

### 3.2. Electroswelling of Lipid

An electroformation chamber was assembled using adhesive tape as illustrated in Figure 1. A thin borosilicate cover glass plate (18 mm × 18 mm, thickness 0.12–0.17 mm), a piece of ITO-PET (16 mm × 20 mm, thickness 0.13 mm) with two rectangular observation windows (typically, 3 mm × 8 mm) as the counter electrode, a polystyrene spacer (20 mm × 20 mm, thickness 1.0 mm), and another piece of the ITO-PET (16 mm × 20 mm) with two small holes (diameter 1 mm) as the formation electrode were stacked in this order. The electroconductive surfaces of the ITO-PET were placed so that they faced each other. Before assembling, eggPC dissolved in methanol (5 mg/mL, 2.0 μL) was spread as a patch of 6 mm × 6 mm on the electroconductive surface of the formation electrode, and the thin lipid layer thus formed was further dried under reduced pressure with a water aspirator. The chamber was filled with Milli-Q grade ultrapure water, and appropriate electric voltage was applied between the electrodes from a function generator (Kenwood TMI FG-272, Yokohama, Japan) in the case of sinusoidal ac or dc pulses. For static dc, electric voltage supplied from dry batteries (6 V) was adjusted by a standard regulator circuit and used. Lipid swelling was observed on an inverted optical microscope equipped with phase contrast and digital image enhancement options (Olympus IX-50, Tokyo, Japan).

In the experiment of electroswelling on ITO-glass, the ITO-PET counter electrode with the slits and the spacer were stacked on an ITO-glass plate (15 mm × 25 mm, thickness 1 mm), on which lipid was deposited. After filling the chamber with ultrapure water through the slits, the thin cover glass plate was placed on the top to seal the chamber.

For the calculation of the GV formation index, the whole area of the deposited lipid was divided into 200 sectors of 200 μm^2^. For each sector, the percentage of the area occupied by spherical GVs larger than 10 μm was determined, and the values were averaged for all the sectors. When GVs were small and larger magnification was needed, smaller sectors were randomly taken (typically, 20–30 sectors of 50 μm^2^) and used in the evaluation. The thickness of the swelling lipid layer was determined by the focal distance in the microscopic observation.

## 4. Conclusions

The present study revealed that electroformation on an ITO-PET electrode occurred as on an ITO-glass electrode. ITO-PET can be a more flexible and less expensive electrode material (see Supplementary Materials for an estimation of the costs) in electroformation. Although in this study, the glass-covered window was made in the counter electrode for the sake of observation with an inverted optical microscope, one may replace it with an intact ITO-PET sheet if only a moderate transparency to visible light, rather than a clear image of GVs, is needed in application. A sensor that outputs photometric change as a primary signal could be such an example. The examination with the three types of electric voltage using ITO-PET electrodes yielded a few valuable insights into electroformation. The mechanical vibration of the swelling lipid layer is not essential for electroformation. Since electroformation occurred with dc voltages, the negative part of ac should mainly drive electroformation. Considering the superior GV formation observed with dc, the positive part of ac could possibly interfere with electroformation.

## Supplementary Files

Supplementary File 1 Supplementary File (PDF, 1546 KB)
