# Supplementary material for: Electroformation of Giant Vesicles on Indium Tin Oxide (ITO)-Coated Poly(ethylene terephthalate) (PET) Electrodes
Source: Membranes (Basel). 2011 May 26;1(2):109–18. doi: 10.3390/membranes1020109 (PMC4021930; doi:10.3390/membranes1020109)
Supplement: Supplementary File 1 — Supplementary File (PDF, 1546 KB) [file membranes-01-00109-s001.pdf]

Supplementary Materials

Fluctuation of membranous objects in the early stage of electroswelling with static dc voltage.

Figure S1. Fluctuation of membranous objects under static dc voltage (3.0 V). The pictures were taken every 30 sec starting from 2 min after the application of the voltage. The length across the frame is 120 μm.

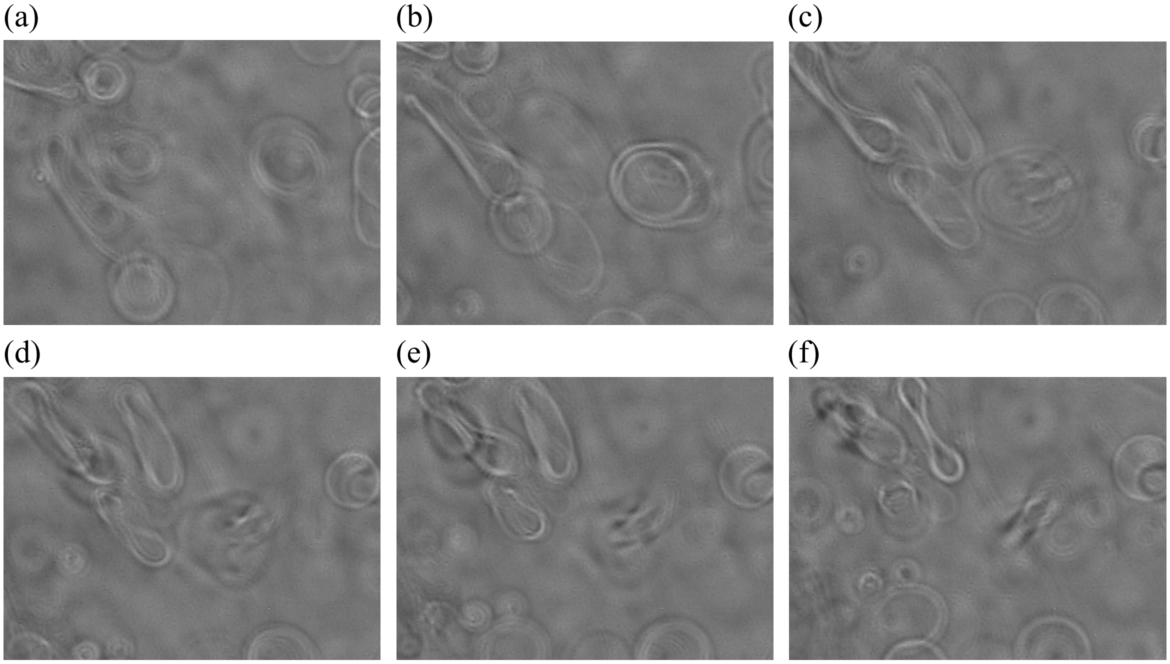

The prices of ITO-PET and ITO-glass.

As of May 11, 2011, Aldrich (http://www.sigmaaldrich.com/) lists the following prices for ITO-PET sheets and ITO-glass plates.

ITO-PET sheets (639281-5ea, 1ft x 1ft, 100 ohm): $78.10/5 sheets

ITO-glass plates (703176-10pak 25 x 25 mm, 70-100 ohm): $35.20/10 plates

In general, ITO-coated materials with lower electric conductivity are less expensive and used in this comparison. The costs can be calculated as $\$0.017/cm^2$ and $\$0.56/cm^2$, for the ITO-PET and ITO-glass, respectively. Although the prices should be different depending on the sources of the materials, in this example, ITO-PET costs approximately 30 times less than ITO-glass.
